# MitoTEMPO Prevents Oxalate Induced Injury in NRK-52E Cells via Inhibiting Mitochondrial Dysfunction and Modulating Oxidative Stress

**DOI:** 10.1155/2017/7528090

**Published:** 2017-01-02

**Authors:** Jiaqiao Zhang, Qing Wang, Chuou Xu, Yuchao Lu, Henglong Hu, Baolong Qin, Yufeng Wang, Deng He, Cong Li, Xiao Yu, Shaogang Wang, Jihong Liu

**Affiliations:** ^1^Department and Institute of Urology, Tongji Hospital, Tongji Medical College, Huazhong University of Science and Technology, Wuhan, Hubei, China; ^2^Department of Radiology, Tongji Hospital, Tongji Medical College, Huazhong University of Science and Technology, Wuhan, Hubei, China; ^3^Department of Gynecology and Obstetrics, Tongji Hospital, Tongji Medical College, Huazhong University of Science and Technology, Wuhan, Hubei, China

## Abstract

As one of the major risks for urolithiasis, hyperoxaluria can be caused by genetic defect or dietary intake. And high oxalate induced renal epithelial cells injury is related to oxidative stress and mitochondrial dysfunction. Here, we investigated whether MitoTEMPO, a mitochondria-targeted antioxidant, could protect against oxalate mediated injury in NRK-52E cells via inhibiting mitochondrial dysfunction and modulating oxidative stress. MitoSOX Red was used to determine mitochondrial ROS (mtROS) production. Mitochondrial membrane potential (Δ*ψ*m) and quantification of ATP synthesis were measured to evaluate mitochondrial function. The protein expression of Nox4, Nox2, and p22 was also detected to explore the effect of oxalate and MitoTEMPO on NADPH oxidase. Our results revealed that pretreatment with MitoTEMPO significantly inhibited oxalate induced lactate dehydrogenase (LDH) and malondialdehyde (MDA) release and decreased oxalate induced mtROS generation. Further, MitoTEMPO pretreatment restored disruption of Δ*ψ*m and decreased ATP synthesis mediated by oxalate. In addition, MitoTEMPO altered the protein expression of Nox4 and p22 and decreased the protein expression of IL-6 and osteopontin (OPN) induced by oxalate. We concluded that MitoTEMPO may be a new candidate to protect against oxalate induced kidney injury as well as urolithiasis.

## 1. Introduction

Hyperoxaluria is considered as one of the primary risk factors for urolithiasis [1]. High oxalate can cause renal tubular cells injury, and this effect is related to reactive oxygen species (ROS) production and imbalance of oxidation-reduction [2]. Mitochondria and NADPH oxidase are the major sources of ROS generation in renal tubular cells, and mitochondrial dysfunction was regarded as a key event in oxalate mediated renal cell injury [3–7].

Various antioxidant treatments including NADPH oxidase inhibitors had been applied to prevent renal cell injury and/or renal crystal formation [8–13]. However, limited research focused on preventing oxalate mediated renal cell toxicity via targeting mitochondria. Further, mitochondria-targeted antioxidant may scavenge mitochondrial ROS (mtROS) without affecting physiological ROS production outside the mitochondria [14].

Targeted therapies for mtROS species have not been reported in oxalate mediated injury in renal tubular cells. MitoTEMPO, a mitochondria-targeted antioxidant can specifically accumulate in the mitochondrial matrix 1000-fold due to its positive charge [15]. We hypothesized that MitoTEMPO prevents oxalate induced injury in renal tubular cell via inhibiting mitochondrial dysfunction and modulating oxidative stress. We hoped to find new and more specific drugs to treat oxalate induced kidney injury as well as urolithiasis.

## 2. Materials and Methods

### 2.1. Reagents

MitoTEMPO was purchased from Sigma-Aldrich (St. Louis, MO, USA) and was dissolved in deionized water according to the molecular weight. Cell counting kit 8 (CCK-8) was purchased from Signalway Antibody LLC (Maryland, USA). LDH cytotoxicity assay kit, 2,7-dichlorodihydrofluorescein diacetate (DCF-DA), JC-1 dye and MDA assay kit, and ATP assay kit were purchased from Beyotime Institute of Biotechnology (Jiangsu, China). MitoSOX Red was purchased from Molecular Probes-Invitrogen (Carlsbad, CA, USA). OPN and Nox4 antibodies were purchased from Abcam (Cambridge, MA, USA); IL6 antibody was purchased from Biorbyt (Cambridge, United Kingdom); p22 antibody was purchased from Santa Cruz Biotechnology (Santa Cruz, CA, USA); Nox2 and *β*-actin antibodies were purchased from Proteintech (Wuhan, China).

### 2.2. Cell Culture

The NRK-52E (normal rat proximal tubular epithelial cell line) was obtained from the Type Culture Collection of the Chinese Academy of Sciences (Shanghai, China). Cells were maintained in Dulbecco's Modified Eagle's Medium (DMEM) (Hyclone; USA) supplemented with 10% fetal bovine serum (Gibco; Grand Island, NY, USA) at 37°C under a humidified 5% CO_2_ atmosphere. Under these conditions, the cells achieved 90% to confluence. They were washed with serum and sodium pyruvate-free DMEM media.

### 2.3. Detection of Cell Viability (CCK-8)

The NRK-52E cells were seeded at the concentration of 2 × 10^3^/well in 96-well plates and incubated overnight. Following pretreatment with or without MitoTEMPO (10 *μ*M) for 1 hour, the cells were treated with or without oxalate (700 *μ*mol/l) for 24 or 48 hours at 37°C. After treatment, 100 *μ*l DMEM media with 10% CCK-8 were added to replace previous media in each well and incubated for another 3 hours. The absorbance at 450 nm was detected. The viability of the cells was recorded as the percentage relative to untreated controls.

### 2.4. Lactate Dehydrogenase (LDH) Assay

The release of LDH into media was measured to detect the injury effect on NRK-52E cells. Cells were seeded in 96-well plates and cultured overnight. Before treatment, they were replaced with serum and sodium pyruvate-free DMEM media. After treatment for various periods, supernatants were collected and detected according to related instruction manual. The LDH release activity was presented as the fold increase over the control group.

### 2.5. Detection of Total Intracellular ROS by Flow Cytometry

Intracellular ROS production was detected using the ROS assay kit as the fluorescence of 2,7-dichlorofluorescein diacetate (DCF-DA) significantly increases after oxidation. Cells were seeded in a 6-well plate and stimulated by oxalate with or without 1 hour of preincubation with MitoTEMPO. After 3 h, cells were treated with DCF-DA (10 *μ*M) for 30 min. Then, the cells were collected and resuspended in 200 *μ*l of PBS buffer. Then intracellular ROS levels were performed by flow cytometry (BD Biosciences) with extinction and emission at 485 and 538 nm. All determinations were performed in triplicate.

### 2.6. Determination of mtROS Levels by Flow Cytometry

MitoSOX Red mitochondrial superoxide indicator was used to determine mtROS production. The cells were treated as the same when detecting intracellular ROS. After treatment, cells were loaded with 5 *μ*M MitoSOX Red reagent for 10 min at 37°C. Then the cells were collected, washed, and resuspended in 200 *μ*l Hanks' balanced salt solution. Flow cytometric analyses were performed with extinction and emission at 510 and 580 nm to determine mtROS. All determinations were performed in triplicate.

### 2.7. Assay of Lipid Peroxidation (MDA)

Lipid peroxidation was measured as malondialdehyde (MDA) content. MDA was determined according to the instructions. The levels of MDA were expressed as percentage relative to the controls.

### 2.8. Detection of Mitochondrial Membrane Potential (MMP) by Fluorescence Microscopy

MMP assay kit (JC-1) was used to detect MMP. Cells were cultured in six-well plates. After stimulation by oxalate with or without 1 hour of preincubation with MitoTEMPO for 3 hours, cells were incubated with dyeing working solution for 20 minutes at 37°C, protected from light. JC-1 selectively entering mitochondria exists as a monomer with green fluorescence (Em 530 nm) at relatively low membrane potential and aggregates with red fluorescence (Em 590 nm) at high membrane potential. Then cells were observed by the fluorescence microscope (Olympus IX73, Japan).

### 2.9. Determination of Cellular ATP

Cellular ATP levels were determined using a commercial ATP assay kit based on the luciferin-luciferase reaction. After treatments, NRK-52E cells were lysed and centrifuged at 12,000*g* for 5 min. Then, 20 *μ*L supernatants were mixed with 100 *μ*L detection working solution in a black 96-well plate. Then, the chemiluminescence was measured. The level of ATP was expressed as percentage relative to the controls.

### 2.10. Western Blotting Analysis

After treatment, the cells were washed and lysed with RIPA buffer that contained protease inhibitor. Same amounts of proteins in each sample were analyzed on 10% SDS-PAGE. After being transferred to PVDF membranes, the membranes were blocked with 3% BSA for 2 h and then the membranes were incubated with primary antibody (1 : 500 for rabbit polyclonal anti-Nox2; 1 : 1,000 for rabbit monoclonal anti-NADPH oxidase 4; 1 : 250 for mouse monoclonal anti-P22; 1 : 1000 for rabbit monoclonal anti-OPN; 1 : 200 for rabbit polyclonal anti-IL6; 1 : 500 for mouse monoclonal *β*-actin) at 4°C overnight; the signals of the membranes were visualized using enhanced chemiluminescence (ECL) after being incubated with secondary antibody (anti-rabbit or anti-mouse IgG) for 1 h at room temperature. Anti-*β*-actin was used to normalize the protein expression.

### 2.11. Statistical Analysis

Results were expressed as the mean ± SEM. One-way ANOVA was used to determine statistically significant differences in these experiments by GraphPad Prism 5 software.

## 3. Results

### 3.1. Effects of Oxalate and MitoTEMPO on NRK-52E Cell Viability

**Figure 1:**
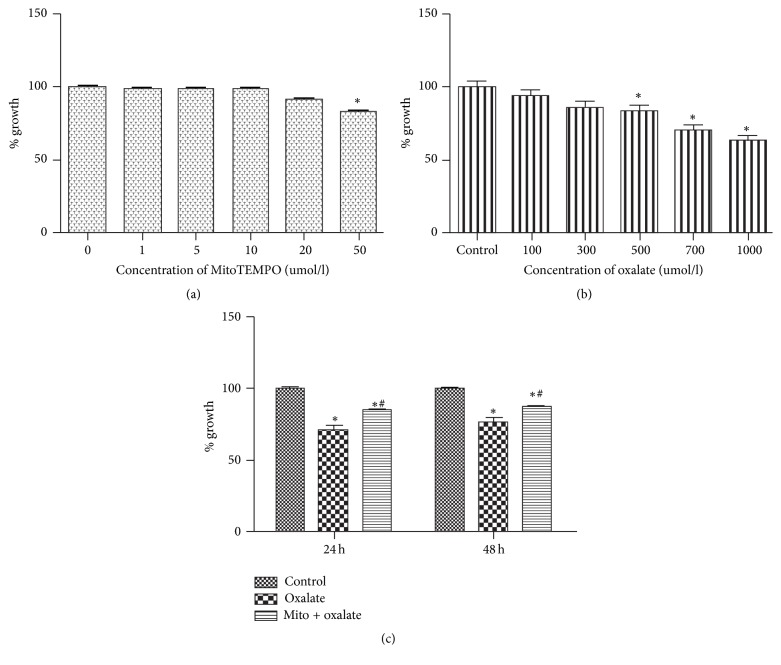
Effects of oxalate and MitoTEMPO on NRK-52E cell viability (a, b, c). The viability of NRK-52E cells was assessed by performing a CCK-8 assay. (a) Cells were treated with MitoTEMPO in different concentrations (0–50 *μ*M). (b) Cells were treated with potassium oxalate in different concentrations (0–1000 *μ*M). (c) MitoTEMPO (10 *μ*M, pretreatment for 1 hour) increased NRK-52E cell viability in the presence of oxalate (700 *μ*M) at 24 and 48 hours. ^*∗*^*P* < 0.05 versus control; ^#^*P* < 0.05 versus oxalate treated group (^*∗*^Mito: MitoTEMPO).

No significant toxicity of MitoTEMPO (1 to 20 *μ*M) was found for NRK-52E cells for 24 hours (Figure 1(a)). The oxalate treatment (for 24 hours) decreased the viability of cells in a dose-dependent manner (Figure 1(b)). According to that, oxalate with concentration of 700 *μ*M was selected for the following experiments. After pretreatment with MitoTEMPO (10 *μ*M) for 1 hour, cells were exposed to oxalate (700 *μ*M) for 24 hours to check cell viability. And we found that treatment with MitoTEMPO could significantly increase the cells viability (Figure 1(c)).

### 3.2. MitoTEMPO Attenuates Oxalate Mediated Cell Injury

**Figure 2:**
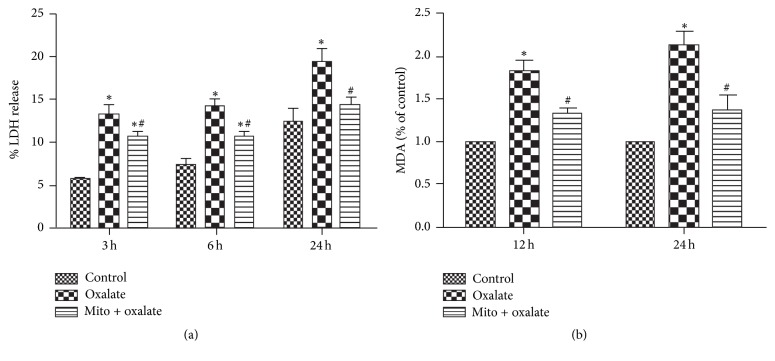
Mito attenuates oxalate induced cell injury and lipid peroxidation (a, b). (a) Mito decreased oxalate induced LDH release at 3, 6, and 24 hours. (b) Lipid peroxidation was assessed by detecting MDA level in the supernatants of NRK cell lysates. Pretreatment with Mito (1 hour) obviously attenuated MDA generation compared with oxalate induced group. ^*∗*^*P* < 0.05 versus control; ^#^*P* < 0.05 versus oxalate treated group (^*∗*^Mito: MitoTEMPO).

To further test the protection of MitoTEMPO against oxalate induced cell injury, the LDH release activity was detected. Exposure of the cells to oxalate (700 *μ*M) resulted in the release of LDH, significantly more than in the controls. In contrast, MitoTEMPO effectively reduced LDH leakage caused by oxalate at different periods (3 to 24 hours) (Figure 2(a)).

### 3.3. MitoTEMPO Reduces Lipid Peroxidation Injury in NRK-52E Cells Exposed to Oxalate

MDA levels were examined as the marker of lipid peroxidation injury and oxidative stress. Treatment with oxalate significantly increased cellular MDA content, and pretreatment with MitoTEMPO significantly decreased the cellular MDA levels compared with oxalate induced cells (Figure 2(b)).

### 3.4. MitoTEMPO Attenuates Oxalate Induced Mitochondrial ROS (mtROS) but not Intracellular ROS Generation

**Figure 3:**
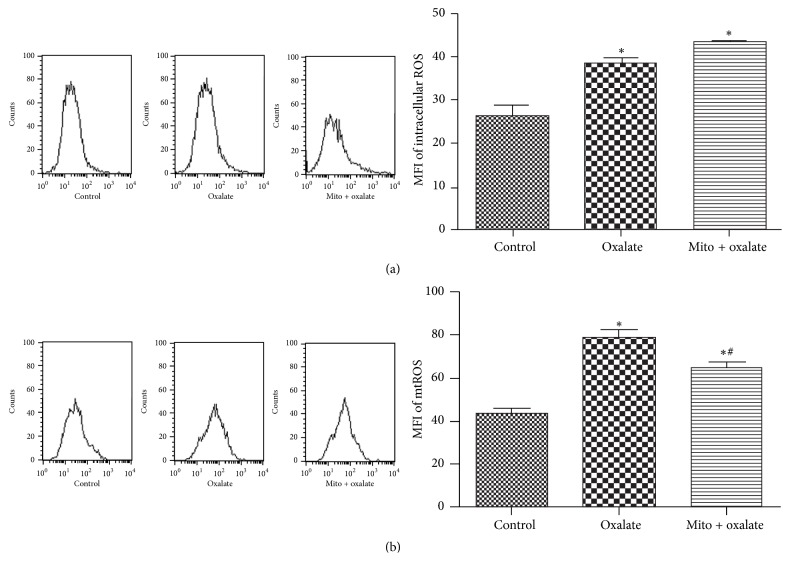
Effect of MitoTEMPO on oxalate induced intracellular ROS and mtROS generation (a, b). NRK-52E cells were stimulated with oxalate (700 *μ*M) for 3 hours with or without preincubation with MitoTEMPO for 1 hour. (a) The intracellular ROS levels were indicated by the fluorescence intensity of DCF analyzed by flow cytometry. (b) The mtROS levels were indicated by the fluorescence intensity of MitoSOX Red analyzed by flow cytometry. Mean ± SEM of three independent experiments. ^*∗*^*P* < 0.05 versus control; ^#^*P* < 0.05 versus oxalate treated group (^*∗*^Mito: MitoTEMPO).

The DCF-DA fluorescence was measured to determine total intracellular ROS level and the intensity of MitoSOX Red fluorescence to determine the mtROS level. Pretreatment with MitoTEMPO significantly decreased the mtROS generation compared with oxalate induced cells; however it had no obvious impact on intracellular ROS generation (Figure 3).

### 3.5. MitoTEMPO Ameliorates Oxalate Induced Disruption of Mitochondrial Membrane Potential

**Figure 4:**
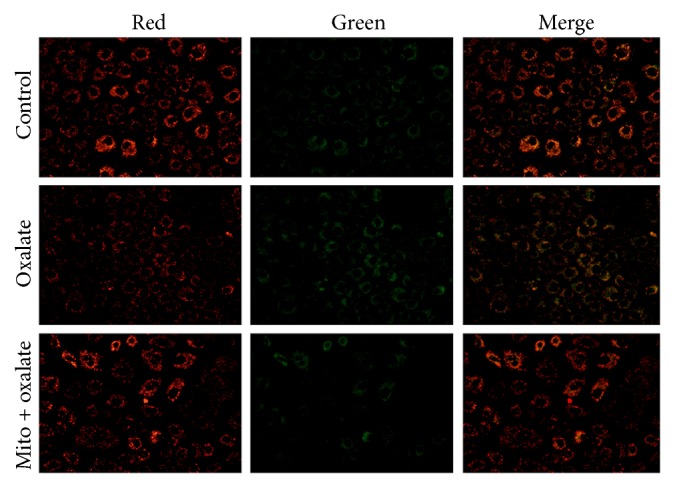
Mito ameliorates oxalate induced disruption of mitochondrial membrane potential. NRK-52E cells were stimulated with oxalate (700 *μ*M) for 1 hour with or without preincubation with MitoTEMPO for 1 hour. Mitochondrial membrane potential was observed using a mitochondrial potential-sensitive dye, JC-1. Cells in control group showed bright red fluorescence. Oxalate treatment attenuated red fluorescence compared with control, and pretreatment with MitoTEMPO reversed these changes (^*∗*^Mito: MitoTEMPO).

Relative to the control group, oxalate exposure produced an obvious reduction in the initial Δ*ψ*_m_, as estimated by the decreased JC-1 fluorescence at 596 nm (red) and concomitant increased fluorescence at 534 nm (green). With MitoTEMPO pretreatment for 1 h, an obvious restoration of MMP was observed (Figure 4). The results indicate that oxalate induced mitochondrial dysfunction, and MitoTEMPO could inhibit this effect.

### 3.6. MitoTEMPO Increases ATP Levels in the NRK-52E Cells Exposed to Oxalate

**Figure 5:**
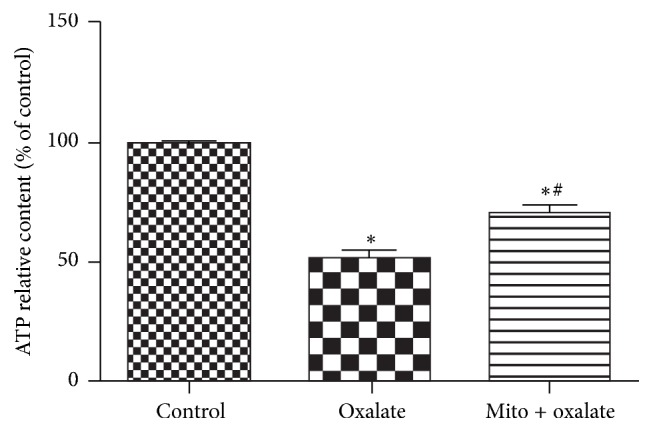
Mito increases ATP levels in the oxalate induced cultured NRK52E cells. NRK-52E cells were stimulated with oxalate (700 *μ*M) for 3 hours with or without preincubation with MitoTEMPO for 1 hour. Oxalate treatment decreased ATP levels significantly. ATP levels in MitoTEMPO pretreatment group significantly increased compared with oxalate treatment group and still was lower compared with control group. ^*∗*^*P* < 0.05 versus control; ^#^*P* < 0.05 versus oxalate treated group (^*∗*^Mito: MitoTEMPO).

Decreased MMP is related to reduction of ATP synthesis. Our results showed that oxalate could induce ATP deficiency in the NRK-52E cells, and MitoTEMPO treatment restored this effect (Figure 5).

### 3.7. MitoTEMPO Selectively Modulated the Protein Expression of NADPH Oxidase Subunits

**Figure 6:**
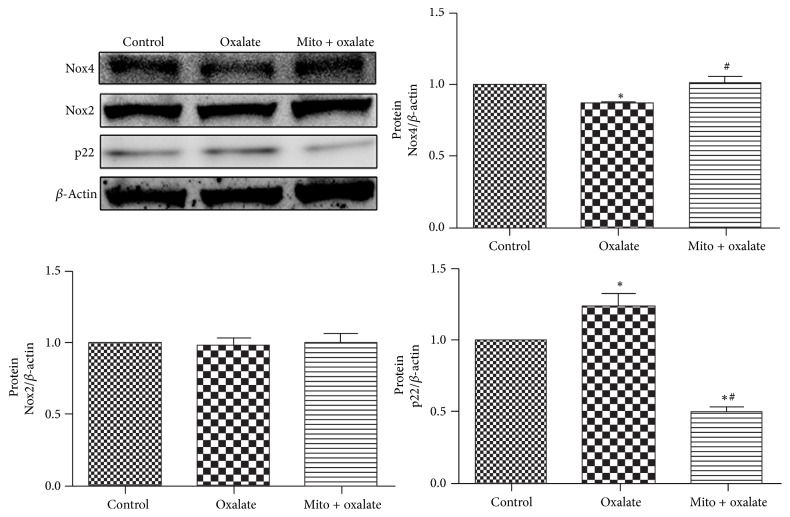
MitoTEMPO selectively modulated the protein expression of NADPH oxidase subunits in NRK-52E cells exposed to oxalate. NRK-52E cells were stimulated with oxalate (700 *μ*M) for 24 hours with or without preincubation with MitoTEMPO for 1 hour. Oxalate treatment decreased the protein expression of Nox4 and elevated the protein expression of p22 significantly compared with the control group. MitoTEMPO pretreatment elevated the protein expression of Nox4 and decreased the protein expression of p22. The protein expression of Nox2 was not changed after oxalate stimulation with or without MitoTEMPO pretreatment. ^*∗*^*P* < 0.05 versus control; ^#^*P* < 0.05 versus oxalate treated group (^*∗*^Mito: MitoTEMPO).

It was reported that oxalate exposure could regulate mRNA expression of NADPH oxidase subunits in a human renal epithelial-derived cell line [5]. In this research, we detected the effect of oxalate and MitoTEMPO on the protein expression of NADPH oxidase subunits. Oxalate treatment decreased the protein expression of Nox4, elevated the protein expression of p22 significantly, and did not change the protein expression of Nox2. MitoTEMPO pretreatment reversed the change of protein expression of Nox4 and p22 without affecting the protein expression of Nox2 (Figure 6).

### 3.8. MitoTEMPO Reduced the Expression of OPN and IL-6 in NRK-52E Cells Exposed to Oxalate

**Figure 7:**
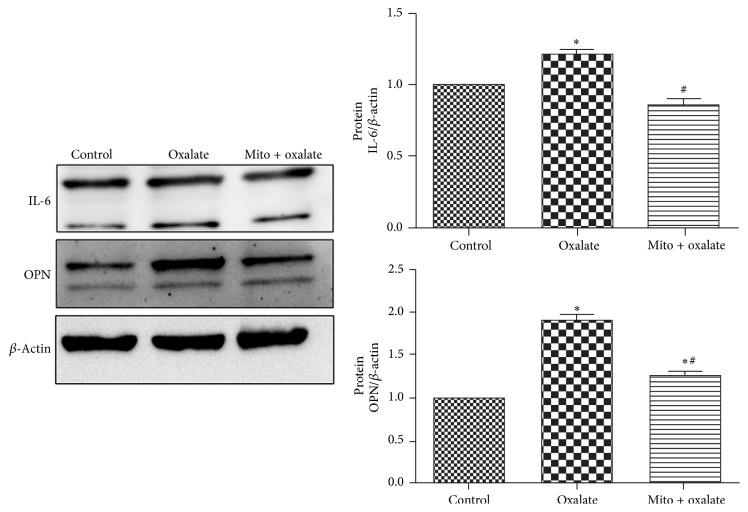
MitoTEMPO reduced the expression of stone modulators of IL-6 and OPN in NRK-52E cells exposed to oxalate. NRK-52E cells were stimulated with oxalate (700 *μ*M) for 24 hours with or without preincubation with MitoTEMPO for 1 hour (^*∗*^Mito: MitoTEMPO).

Oxalate significantly increased the protein expression of OPN and IL-6. Furthermore, the increased expression of OPN and IL-6 was significantly attenuated when cells were pretreated with MitoTEMPO (Figure 7).

## 4. Discussion

Numerous studies have indicated that ROS and oxidative stress were involved in the stone formation as well as oxalate induced renal epithelial cells injury. As two important sources of ROS production, both mitochondria and NADPH oxidases were involved in oxalate induced renal epithelial cells injury [3, 6, 13, 16, 17].

However, ROS generation is not always detrimental, as it is involved in both physiological and pathological processes. Many intervention trials with antioxidants supposed to prevent oxidative stress and improve disease outcomes have been mostly invalid or harmful. An important reason for these failures may be attributed to inappropriate inhibition of physiological ROS [18]. Therefore, theoretically we should intervene by targeting pathological ROS while making physiologically relevant ROS unaffected. Interestingly, it was reported that mtROS could directly contribute to inflammatory cytokine production and we suggested that mtROS may be a type of pathological ROS [19, 20]. Based on these evidences, we propose a hypothesis that MitoTEMPO, a mitochondria-targeted antioxidant, would exert a protective effect against oxalate induced renal epithelial cells injury via specifically acting on mitochondria and modulating ROS generation.

In our study, MitoTEMPO significantly increased the cells viability following exposure to oxalate. Also pretreatment with MitoTEMPO obviously decreased NRK-52E cells injury assessed by LDH release. The marker of oxidative stress, MDA level, was also decreased in MitoTEMPO-treated group. We also demonstrated that MitoTEMPO increased the cells viability via inhibiting mitochondrial ROS generation and restoration of mitochondrial membrane potential as well as promoting ATP synthesis.

Interestingly, our research found that MitoTEMPO attenuated mtROS generation caused by oxalate stimulation but not intracellular ROS generation. This phenomenon may be because MitoTEMPO can selectively accumulate in the mitochondria. Of course, there may be interaction between mitochondria and NADPH oxidase. Research indicated that oxidative stress provokes a positive-feedback loop between the two, although exact mechanism is undefined [15, 21]. As previous studies have indicated oxalate could activate the NADPH oxidase and upregulate the expression of some NADPH oxidase subunits [5, 17], we wondered if NADPH oxidase subunits could be regulated by MitoTEMPO treatment. And we observed that the protein expressions of Nox4 and p22 were also changed after oxalate and oxalate and MitoTEMPO combination treatment. This result also indicated mtROS may regulate NADPH oxidase activity via regulating the protein expression of NADPH oxidase subunits although we did not detect the activity of the NADPH oxidase.

The family of NADPH oxidases consists of seven isoforms (Nox1–5, Duox1, and Duox2). Of the seven isoforms, Nox4 and Nox2 are the two major components in the kidney, and activation of both Nox4 and Nox2 depends on p22 [22]. In contrast to NADPH oxidase Nox2, Nox4 produces hydrogen peroxide rather than superoxide [23]. Based on these evidences, we speculate that MitoTEMPO may play a protective role in oxalate mediated cell injury through modulating not only “dose” but also “type” of ROS generation.

As one of the proinflammatory cytokines, IL-6 may be involved in pathogenesis of urolithiasis. It was reported that patients of urolithiasis were associated with elevated level of IL-6 [24]. Study also indicated that oxalate could increase the expression of IL-6 in a time- and concentration-dependent manner [25]. As publications have discovered that mtROS acted as signaling molecules to lead inflammatory cytokine production [26–28], we determine the effect of oxalate and MitoTEMPO on the expression of IL-6. And our results demonstrated that oxalate also upregulated the expression of IL-6 in NRK-52E cells, and MitoTEMPO treatment could weaken this effect.

Osteopontin (OPN), secreted by renal tubular epithelial cells, is considered as an important macromolecular modulator in the development of urolithiasis. The OPN expression is upregulated in both hyperoxaluric rats and genetic hypercalciuric rats [29, 30]. Studies also revealed that high concentration of calcium, oxalate, or calcium oxalate monohydrate (COM) crystals could stimulate OPN expression in renal tubular cells [4, 31, 32]. In our study, MitoTEMPO modulated ROS (including mitochondrial and intracellular) generation and OPN expression induced by oxalate. It is consistent with previous reports that OPN expression is regulated by ROS [4, 33].

There are some limitations in our study. Firstly, we just determined a part of NADPH oxidase subunits and did not include NADPH oxidase activity. Secondly, we did not detect specific type of intracellular ROS, for example, hydrogen peroxide or superoxide anion. At last, how MitoTEMPO affects downstream redox signaling such as p38, JNK, and ERK1/2 pathways is yet to be known.

In our study, MitoTEMPO may play a role to inhibit “harmful” ROS to confer protection against oxalate induced cell injury. Furthermore, MitoTEMPO decreased protein expression of IL-6 and OPN which are sensitive to redox regulation. To our knowledge, it is the first time to apply mitochondria-targeted antioxidant to inhibit oxalate induced cytotoxicity. Our present study implicates that MitoTEMPO may be an ideal candidate to control oxalate induced kidney injury as well as urolithiasis.
